# Embedding of Molecular Structure Using Molecular Hypergraph Variational Autoencoder with Metric Learning

**DOI:** 10.1002/minf.202000203

**Published:** 2020-11-23

**Authors:** Daiki Koge, Naoaki Ono, Ming Huang, Md. Altaf‐Ul‐Amin, Shigehiko Kanaya

**Affiliations:** ^1^ Division of Information Science Graduate School of Science and Technology Nara Institute of Science and Technology 8916-5 Takayama, Ikoma Nara 630-0192 Japan; ^2^ Data Science Center Graduate School of Science and Technology Nara Institute of Science and Technology 8916-5 Takayama, Ikoma Nara 630-0192 Japan

**Keywords:** chemical space, molecular hypergraph, metric learning, variational autoencoders

## Abstract

Deep learning approaches are widely used to search molecular structures for a candidate drug/material. The basic approach in drug/material candidate structure discovery is to embed a relationship that holds between a molecular structure and the physical property into a low‐dimensional vector space (chemical space) and search for a candidate molecular structure in that space based on a desired physical property value. Deep learning simplifies the structure search by efficiently modeling the structure of the chemical space with greater detail and lower dimensions than the original input space. In our research, we propose an effective method for molecular embedding learning that combines variational autoencoders (VAEs) and metric learning using any physical property. Our method enables molecular structures and physical properties to be embedded locally and continuously into VAEs’ latent space while maintaining the consistency of the relationship between the structural features and the physical properties of molecules to yield better predictions.

## Graphical abstract

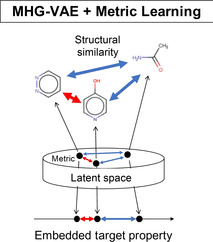

Molecular design aims to identify molecular structures with certain desirable properties. However, the search space for a target molecular structure that is a candidate drug or useful material is too complicated and it is difficult to search for desirable molecules. Properties of complex organic molecules are often represented by a wide range of descriptors so that the molecules can be embedded into a multidimensional space called a chemical space.^[1]^ However, closeness in a descriptor space does not always imply similarity in molecular structures. Creating a chemical space that reflects the similarity in the physical properties as well as molecular structures will drastically improve the efficiency of molecular design. And a wide diversity of molecular property in the dataset is important to generalize prediction.^[2]^ In this paper, we introduce a model to embed molecules into a latent space using deep learning to reproduce the distance between the properties of chemicals based on their molecular structures.

We apply a deep learning model to build an encoder from a molecular formula in a latent space and trained the model using a publicly available dataset for the multiple physical properties of organic molecules named QM9.^[3]^ We introduce a training scheme based on the metric learning method to maintain the consistency of neighboring molecules in the latent space, which should show similar physical properties, such as chemical potential (i. e., internal energy), potential of the highest orbit, and heat capacity.

Gómez‐Bombarelli and others[4] used a variational autoencoder (VAE)^[5]^ with simplified molecular input line entry system (SMILES)^[6]^ character strings as input. Moreover, they imposed a constraint on the learning of VAE by jointly training a physical property linear regression model. A regression model that predicts a physical property value from a latent vector with VAE and VAE joint training with large amounts of labeled data makes VAE latent space organized based on the physical property. By using a Bayesian optimization (BO)^[7]^ search on the latent space based on a physical property value, we can identify molecular structures that have a certain desirable property as SMILES sequences using Recurrent neural networks (RNN). However, because the latent space of the VAE that has learned the SMILES character string is composed of the sequential features of SMILES, the learned latent space does not configure the latent space that properly embeds the features of the molecular structure.^[8]^ Kajino^[9]^ developed a hypergraph grammar‐based method for molecular structure generation. In this method (molecular hypergraph grammar variational autoencoder (MHG‐VAE)), a molecular structure is described as a hypergraph, and Grammar VAE^[10]^ is trained by inputting the production rule sequence of the hypergraph. MHG‐VAE can embed latent features of molecular structures into the VAE latent vector more precisely than VAEs that used SMILES and junction tree^[11]^ as molecular descriptors. The molecular structure design models using these VAEs commonly have the VAE latent space organized based on specific physical properties by joint learning with the physical property linear regression model. However, existing joint learning with regression (Joint VAE) has a problem in embedding physical properties from the perspectives of the loss function and the learning method.

The objective function of VAEs is given as follows:

(1) $${𝔼}_{z∼q_{\phi \left(z\right|x)}}\left[log\;p_{\theta }\left(x|z\right)\right]-D_{KL}\left(q_{\phi }\left(z|x\right)∥p\left(z\right)\right),$$

where z is a latent vector in which input data x is encoded by VAEs Encoder $q_{\phi }\left(z|x\right)$ . ϕ is a parameter of VAEs Encoder. $p_{\theta }\left(x|z\right)$ is VAEs Decoder. θ is a parameter of VAEs Decoder. VAEs minimize the following loss function of Eq. (2) by optimizing the parameters ϕ and θ to maximize the objective function of Eq. 1:

(2) $${ℒ}_{\mathrm{VAE}}={ℒ}_{\mathrm{Reconstruction}}+{ℒ}_{\mathrm{KL}\;\mathrm{divergence}},$$

where the ${ℒ}_{\mathrm{Reconstruction}}\;$ term is given by the cross entropy of the input data vector x to the VAEs Encoder and the reconstructed vector is given by the VAEs Decoder. ${ℒ}_{\mathrm{KL}\;\mathrm{divergence}}$ is the KL divergence between the approximate posterior distribution of latent vectors obtained by the VAEs Encoder and a prior distribution $p\left(z\right)$ (Standard normal distribution). Additionally, Joint VAE minimizes the loss function given by the Eq. (3) by adding a regression for the loss ${ℒ}_{\mathrm{reg}\;\mathrm{loss}}$ term to Eq. 2:

(3) $${ℒ}_{\mathrm{Joint}\;\mathrm{VAE}}={ℒ}_{\mathrm{Reconstruction}}+\beta {ℒ}_{\mathrm{KL}\;\mathrm{divergence}}+\;{\gamma ℒ}_{reg\;loss},$$

where,

$${ℒ}_{\mathrm{reg}\;\mathrm{loss}}=\;\frac{1}{N}\sum_{i=0}^{N}{(f(z_{i})-\;y_{i})}^{2},$$

where $f\left(z_{i}\right)$ denotes the predicted value of the physical property regression model corresponding to the *i*‐th input data, and y is the true value. β and γ are hyperparameters. Linear regression $f\left(z_{i}\right)\;$ predicts a physical property value from a latent vector $z_{i}$ corresponding to the *i*‐th input data $x_{i}$ . A linear regression optimizes its parameters to minimize ${ℒ}_{\mathrm{reg}\;\mathrm{loss}}\;$ . Joint VAE encoder maps an input data $x_{i}$ to the latent vector $z_{i}$ so that ${ℒ}_{\mathrm{reg}\;\mathrm{loss}}$ becomes small. Therefore, Joint VAE latent space becomes organized based on a physical property that is used for regression. However, Joint VAE may embed linearly a target physical property value into very few axes of the latent vector *z*. For example, Joint VAE latent vector *z* has *D* dimensions, and If one of the *D* latent variables can sufficiently express the physical property, this physical property value would be linearly embedded in one variable. Such representation of the latent vector is not preferable because molecules that have similar physical properties in a few descriptors do not necessarily have similar structures. Therefore, when we output molecular structures based on the target property value embedded from the latent space of Joint VAEs, structurally highly dissimilar molecules may be output (Figure 1, Joint VAE). Also, the output molecules most likely have large variations in other properties other than the targeted property. Such large variations in some properties are not suitable for designing a target molecule such as a ligand molecule in which several physiological/biological properties (toxicity, water solubility, and binding affinity) should be carefully adjusted. Conversely, if VAEs with a molecular descriptor as the input are trained without any constraint (physical property regression), the specific physical property values may not be continuously embedded. Therefore, if molecular structures are continuously selected from the neighboring points on the VAE latent space, a target physical property may not change continuously (Figure 1, VAE only). To address this dilemma, we need to continuously embed the molecular structure into the VAE latent space while maintaining the consistency of the relationship between molecular structure and physical properties.

**Figure 1:**
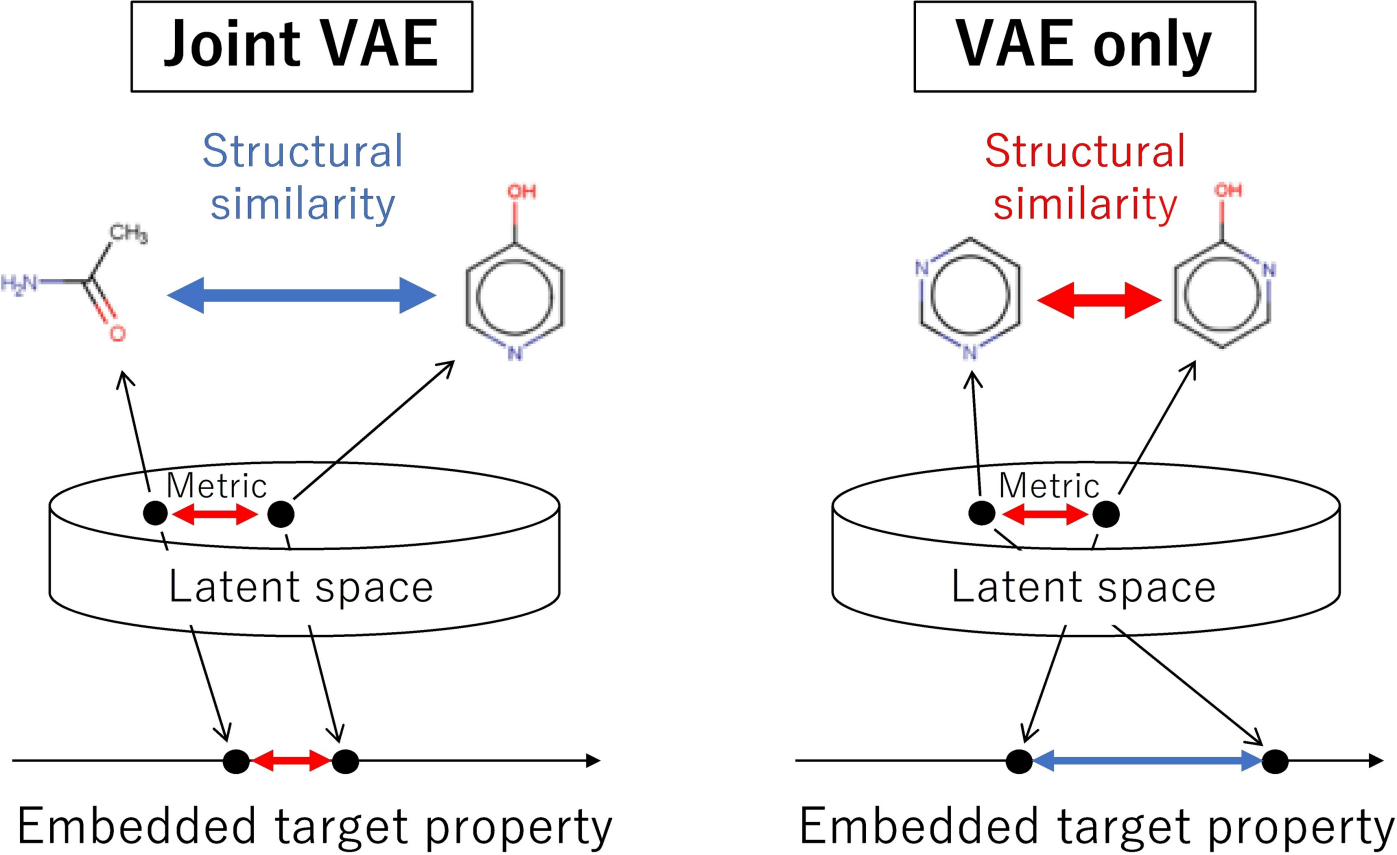
Schematic of VAE latent space in the existing methods. Neighboring molecules in the latent space are not always show similar structures or similar target properties.

To address the problem, we applied metric learning into a drug/material design model using VAEs. Metric learning is a learning method that matches a distance similarity in a label space with a distance similarity in an embedding space by a neural network. In this study, we propose a learning method that combines log ratio loss^[12]^ with the loss function of VAE Eq. (2). Log ratio loss can handle similarity defined by continuous labels. The model of our proposed method is shown in Figure 2. The VAE architecture uses the same model as Kajino's MHG‐VAE^[9]^ because MHG‐VAE can embed latent features of molecular structures into the VAE latent vector more precisely than VAEs that use SMILES and other descriptors. At first, we extract some molecular hypergraph grammar sequences from a molecular data set using a molecular hypergraph grammar inference algorithm and convert molecules into the production rule sequences using the extracted hypergraph grammar. MHG‐VAE is trained with production rule sequences as input. Our model (Metric VAE) optimizes neural network parameters by minimizing loss function given in Eq. (4), and the ${ℒ}_{\mathrm{Reconstruction}}$ and ${ℒ}_{\mathrm{KL}\;\mathrm{divergence}}$ are the same terms as the loss function of MHG‐VAE, while the ${ℒ}_{\mathrm{Reconstruction}}\;$ term is a cross entropy of an input sequence vector (production rule sequence) to the MHG‐VAE encoder and a reconstructed sequence vector by the MHG‐VAE decoder. ${ℒ}_{\mathrm{KL}\;\mathrm{divergence}}$ is the KL divergence between an approximate posterior distribution of latent vectors obtained by the MHG‐VAE encoder and a prior distribution (Standard normal distribution). MHG‐VAE continuously embeds a molecular structure into the VAE latent space. However, it is difficult for MHG‐VAE training without any constraint to continuously embed a targeted physical property value into the MHG‐VAE latent space. Eq. (4) ${ℒ}_{\log\;\mathrm{ratio}\;\mathrm{loss}}$ term is a loss term that imposes a constraint so that a physical property value is embedded locally and continuously into the MHG‐VAE latent space. The β and γ are hyperparameters. The ${ℒ}_{\log\;\mathrm{ratio}\;\mathrm{loss}}$ term is calculated by Eq. 5.

(4) $${ℒ}_{\mathrm{Metric}\;\mathrm{VAE}}={ℒ}_{\mathrm{Reconstruction}}+\beta {ℒ}_{\mathrm{KL}\;\mathrm{divergence}}+{\gamma ℒ}_{\log\;\mathrm{ratio}\;\mathrm{loss}},$$

**Figure 2:**
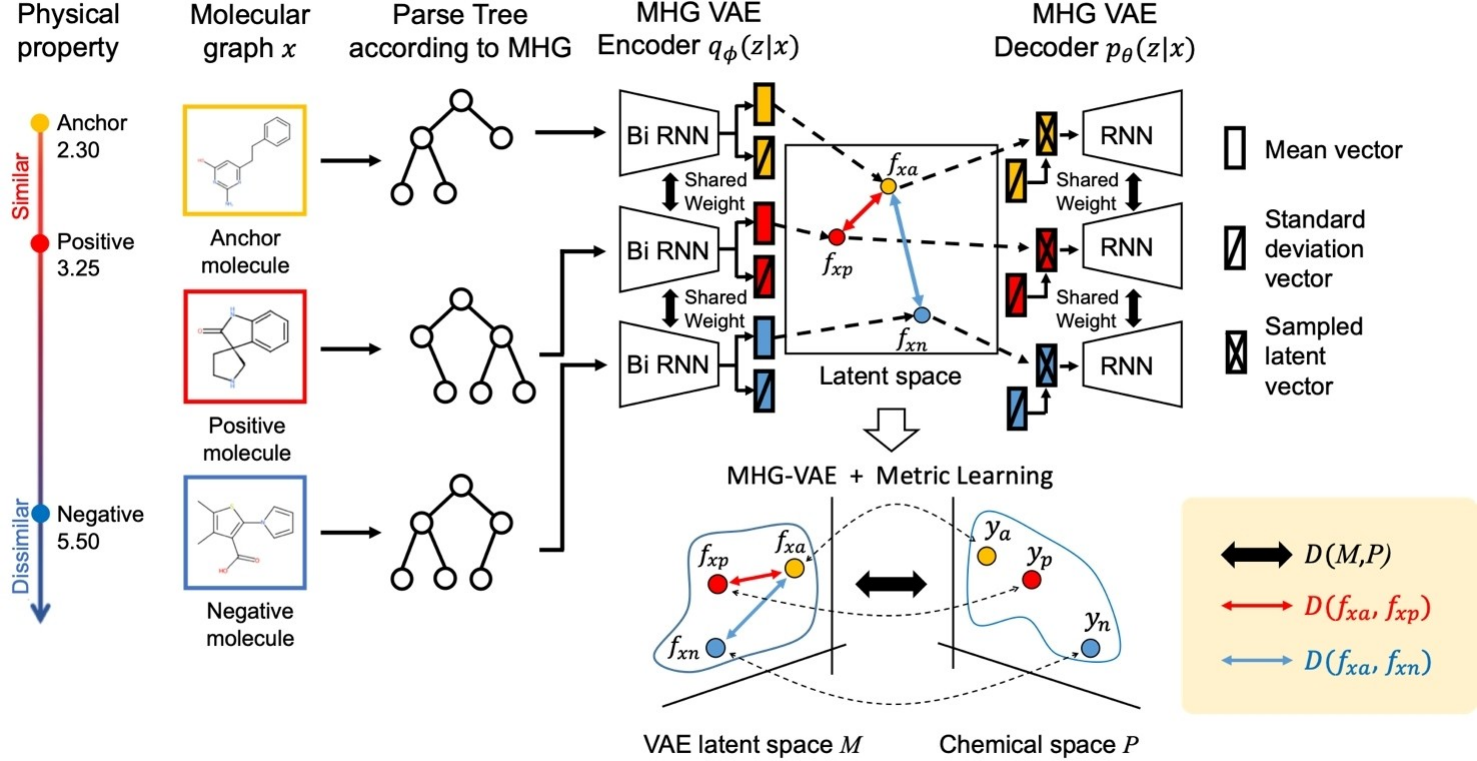
Model schematic. To calculate metric loss, we label molecules with anchor, positive, and negative in molecules data set. In this figure, *a*, *p*, and *n* represent anchor, positive, and negative samples, respectively. $f_{x}$ is a latent vector corresponding to the input molecular graph x . The VAE architecture uses the same model as Kajino's MHG‐VAE composed of encoder using Bi‐directional RNN and decoder using RNN. By optimizing the positions of the anchor, positive, and negative latent vectors, VAE latent space *M* becomes closer to the Chemical space *P*. D(X,Y) represents the distance between X and Y .

where,

(5) $$\;{ℒ}_{\log\;\mathrm{ratio}\;\mathrm{loss}}=\;{\left\{\log\frac{D(f_{xa},f_{xn})}{D(f_{xa},f_{xp})}\;-\;\log\frac{D(y_{a},y_{n})}{D(y_{a},y_{p})}\right\}}^{2},$$

where $f_{x}$ is a latent vector corresponding to an input molecular graph x , *y* is a physical property, and *D*(^.^) denotes squared Euclidean distance. *a*, *p*, and *n* represent anchor, positive, and negative samples, respectively. Positive and negative samples are selected by distance to the anchor sample in the target physical property. The triplet‐based metric learning calculates a loss value using a sampling method called triplet sampling based on the label distance from such a data sample.^[13]^ By approximating the ratios between label distances instead of the distances themselves, the proposed loss enables the learning of a metric space more flexibly regardless of the scale of the physical property values. We extract three samples required for the calculation of ${ℒ}_{\log\;\mathrm{ratio}\;\mathrm{loss}}$ from batch data sample by triplet sampling based on any physical property value and calculate the log ratio loss for each batch of data samples. However, calculating ${ℒ}_{\log\;\mathrm{ratio}\;\mathrm{loss}}$ for all samples in a batch tensor during batch training requires huge computational cost, and metric loss by triplet sampling focusing only on a physical property value used for learning may compete with the MHG‐VAE training. To carefully handle the closeness of structural features of molecules and the closeness of the physical property values used for metric learning, our method does not treat samples that are too far apart in the MHG‐VAE latent space as positive and negative samples for calculation of the ${ℒ}_{\log\;\mathrm{ratio}\;\mathrm{loss}}$ term. We show an illustrative example of our proposed triplet sampling for the continuous embedding of molecular structure in Figure 3. At first, we select a sample to be set as an anchor in a batch tensor and sort the batch samples based on MHG‐VAE latent space distance (Euclidean distance). Second, we sort 25 % of the samples based on a specific physical property distance (Euclidean distance). Lastly, we select positive and negative samples in turn from sorted samples based on physical properties. We repeat steps 1 to 3 while changing the index of the anchor selected in step 0 (Figure 3).

**Figure 3:**
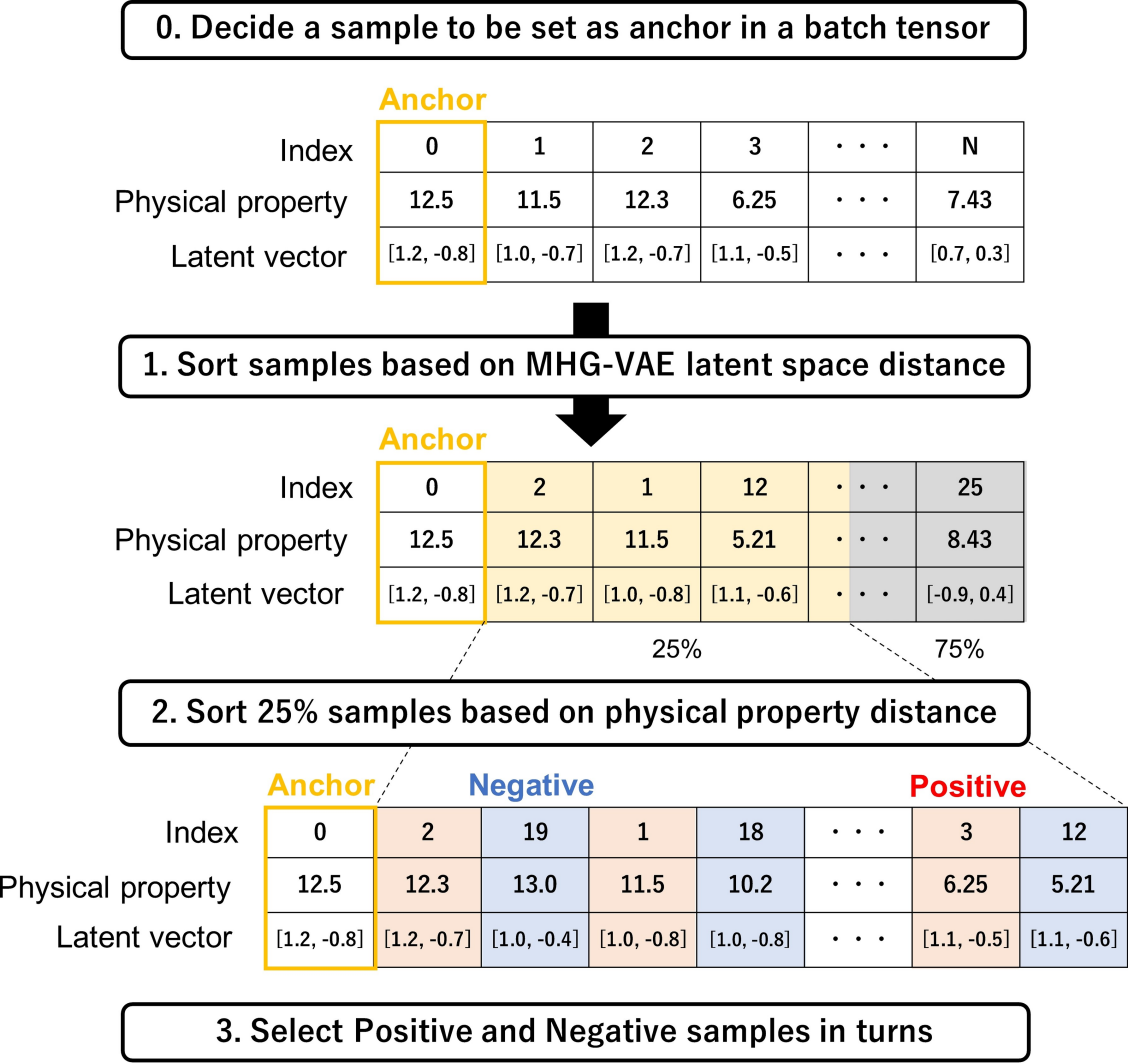
Triplet sampling for calculating log ratio loss. This is an example overview of the triplet sampling method when the VAE latent vector is two‐dimensional.

Metric learning based on log ratio loss can be integrated by the training of normal VAEs. In addition, our proposed method can circumvent the problem that the variation of a property is limited to only a few dimensions in the latent space because it is necessary to adjust all the variables of a latent vector and to embed the property locally and continuously in the MHG‐VAE latent space to reduce the ${ℒ}_{\log\;\mathrm{ratio}\;\mathrm{loss}}$ term. Therefore, we can embed molecular structures and physical properties locally and continuously in the VAE latent space while maintaining the consistency of the relationship between the structural features and the physical properties of molecules (Figure 4).

**Figure 4:**
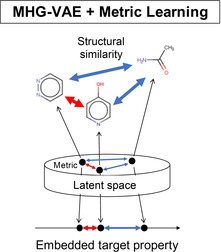
Schematic of VAE latent space in our proposed method. The correspondence between structural features and property value is consistently aligned in latent space.

Moreover, when we output molecular structures based on the targeted physical property embedded from the MHG‐VAE latent space of our proposed model, structurally similar molecules will be determined (Figure 4).

All deep neural network architectures build a representation of the input data in the middle layer. Our model can save the middle layer activation of Metric VAE as a fixed dimension continuous local embedding of physical properties. Using the embedding technique for a low‐dimensional vector by deep learning results in the higher quality of the vector expression, and it works more effectively for a specific task.^[14]^ For instance, ImageNet embeddings are often used as‐is to make predictions on unrelated image tasks.^[15]^ In recent years, such an embedding learning method has been applied not only in the field of natural language processing and image analysis, but also in the field of computational chemistry.^[16]^

From approximately 130 K data (QM9), we randomly selected 80 % as training and 20 % as test data sets. We evaluated the embedding space extracted by the existing method (Joint VAE) and our proposed method as a physical property regression problem using neighbor points in VAE latent space. We started by constructing two VAE models, VAE that was jointly learned with a regression model and VAE with Log ratio loss (our proposed model), to encode a molecular structure into a continuous latent vector representation. We propose an evaluation method using neighboring points on the embedding space to quantitatively evaluate the representation of the VAEs embedding space (Figure 5). First, we extracted embedding vectors from each of the 1,000 molecular data randomly selected from hold out which is not included test data using a VAE encoder. Second, we extracted neighbor points that correspond to the training data embedded by the same procedure as the first procedure in the MHG‐VAE embedding space. Finally, we constructed a physical property linear regression model using only ten neighbor points, and we calculated the mean absolute error (MAE) when predicting the physical property value of the molecule (located at the center) of validation data. Lower MAE scores for the physical properties not used for embedding learning, as well as the scores which were used in the learning, imply that the structural features and the physical properties of molecules are embedded in the MHG‐VAE space locally and continuously. We reported MAE scores when performing embedding learning with three different physical property values using the existing method (Joint VAE) and our proposed method (Metric VAE) in Figure 5. The $U_{0}$ (internal energy at 0 K), U (internal energy at 298.15 K), G (free energy), and H (enthalpy) have strong linear correlations; thus, we summarized the four physical properties in the $U_{0}$ descriptor. As shown in Table 1, Metric VAE outperforms Joint VAE on nearly all QM9 descriptors when selecting each of the three physical property values ($U_{0}$ , Cv, and Highest occupied molecular orbital (HOMO)) as the embedding learning label. The properties of each symbol are shown in Table 2. We selected the three properties which have high variances as the label of embedding learning.

**Figure 5:**
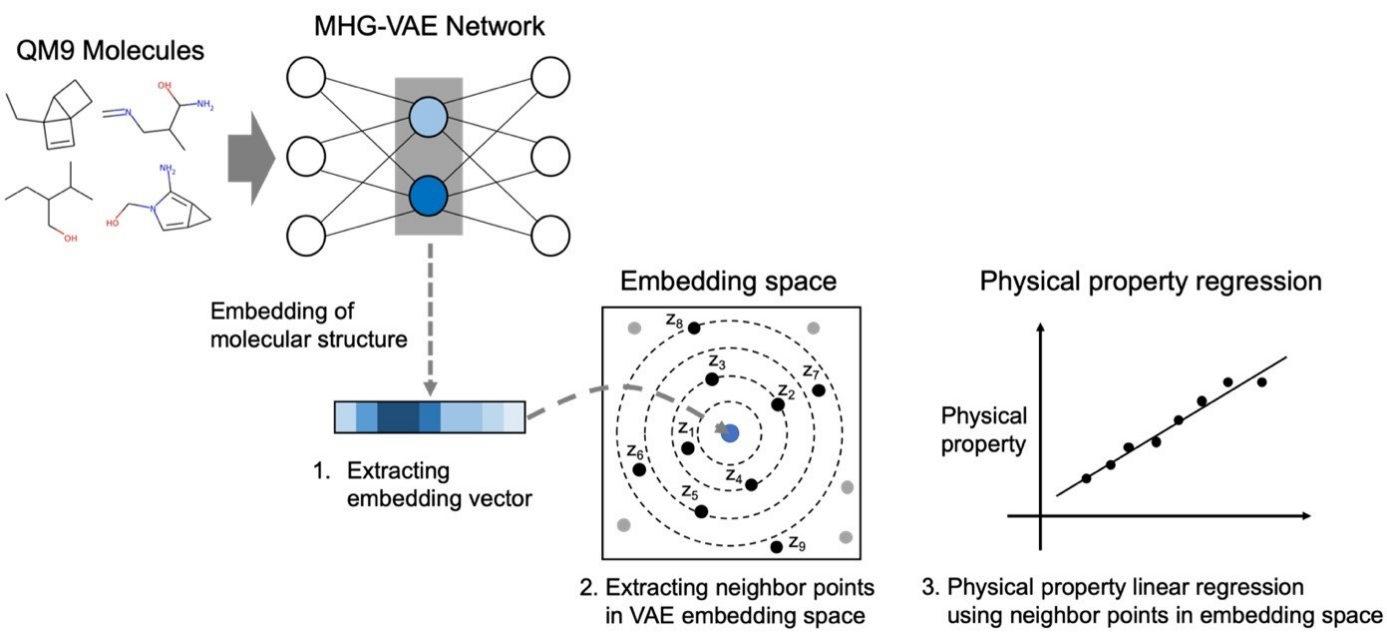
An overview of the evaluation method. The outputs from the middle layer of VAE is reduced to a vector, which is used for predicting physical properties. The number z_i_ described in the embedding space represents a neighboring point in the embedding space for the embedding vector and represents the order of proximity.

**Table 1:** Comparison of MAE with the models for QM9 data sets. The numbers highlighted in bold show that the model is better. The label indicates the physical property used for embedding learning. Joint and Metric represent the existing and proposed methods, respectively. In our method (Metric), the improvement of the prediction of target property does not have much influence on the prediction of other physical properties.

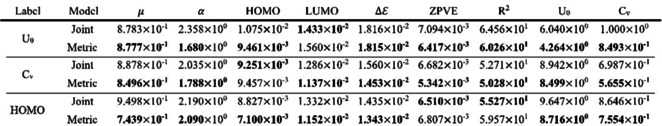

**Table 2:** Target properties of the QM9 data set.

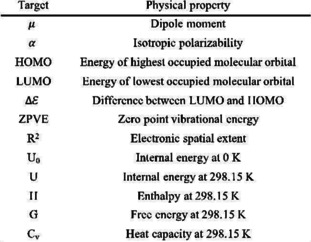

To compare Joint and Metric VAEs, we calculated the accuracies of two models in regression using the 203 RDKit^[17]^ descriptors (Figure 6). We extracted physical property descriptors having continuous values and nonzero variance. Consequently, we evaluated the models using 83 physical property values (The list of all 83 properties of RDKit descriptors are shown in the section S1 of the Supplemental information). We plotted the MAE scores according to the models used for embedding learning and the three physical property values. The y‐axis represents MAE calculated by Joint VAE, and the x‐axis represents MAE calculated by Metric VAE. Figure 6 shows that Metric VAE shows overall lower MAE scores than Joint VAE. Metric VAE outperforms Joint VAE on 165 out of 249 evaluation points (60 (72.2 %) for $U_{0}$ , 53 (63.3 %) for Cv, and 52 (62.6 %) for HOMO respectively). This result implies that our approach can be extended to a wide range of chemical descriptors related with basic physical properties. However, note that this approach is difficult to apply for descriptors and properties which are not correlated with the molecular features embedded as target properties.

**Figure 6:**
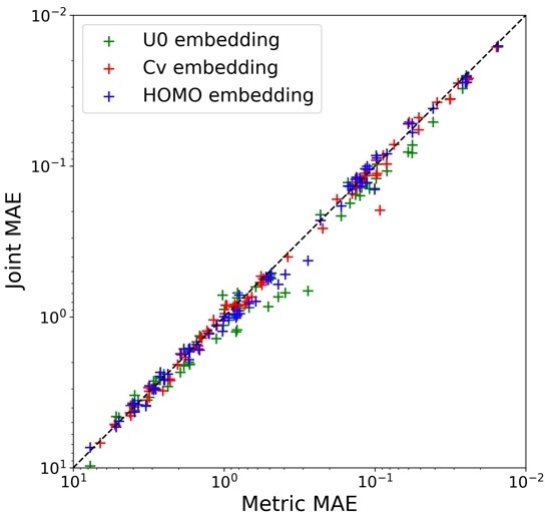
MAE score comparison of Joint and Metric VAEs (our proposed model) broken down by RDKit descriptors and embedding labels. Each dot represents RDKit descriptors, while each marker color represents a physical property value used in the embedding learning model.

Moreover, Metric VAE underperforms Joint VAE for some descriptors; however, the difference is small and for some descriptors, it improves the prediction significantly compared to Joint VAE. Therefore, we successfully extracted the latent vector representation in which physical property values and structural features of a molecule are smoothly embedded from the molecular structure compared with the conventional methods. When we optimize molecular structure based on a target property by Metric VAE, we can avoid the problem which other properties will be secondarily greatly changed. The advantage of metric learning is based on local consistency of the embedded chemical space, so this approach would be applicable for other datasets of natural metabolic compounds. Since these results also suggest that our proposed method can model a chemical space in which more physical property values are smoothly embedded as the number of types of physical property values used for embedding learning increases, a future research direction is to model an ideal chemical space for drug design by transforming the MHG‐VAE latent space using our proposed method as transfer learning.

## Computational Methods

To show that our method is effective for molecular structure design using molecular data analysis, we applied the models (Joint and Metric VAEs) to a public chemical dataset. Since we are interested in comparison of multiple physical properties, we chose QM9 dataset. The QM9 data set contains approximately 130 K examples of stable small organic molecules with up to nine heavy atoms (C, O, N, and F). The 12 target properties for each example are shown in Table 2. All properties are calculated at the B3LYP/6‐31G (2df, p) level of quantum chemistry. We extracted 807 molecular hypergraph grammar sequences from the QM9 data set using molecular hypergraph grammar and converted approximately 130 K molecules into production sequences with a maximum length of 12. Both the VAE encoder and decoder use three‐layer GRU^[18]^ with 384 hidden sizes (encoder is bidirectional), handling a sequence of production rules embedded in 900‐dimensional space. In the encoder, the output of the GRU is fed into a linear layer to compute the mean and log variance of a 50‐dimensional Gaussian distribution, and the latent vector *z* $\in {\mathbb{R}}^{50}$ is sampled from it as the output of VAE encoder. The VAE objective is optimized with Adam,^[19]^ and the learning rate is 0.001.

To prevent the problem of not reducing the reconstruction error due to the influence of all parameters of the latent variable on the embedded properties during the early steps of learning, we employ *β*‐TCVAE.^[20]^ The loss function of *β*‐TCVAE is given as follows:

$${ℒ}_{\mathrm{beta}-\mathrm{TCVAE}}={ℒ}_{\mathrm{Reconstruction}}+{\alpha_{1}D}_{KL}\left(q_{\phi }\left(z,n\right)∥q_{\phi }\left(z\right)p\left(z\right)\right)+\;{\alpha_{2}D}_{KL}(q_{\phi }(z)∥\prod_{j}q_{\phi }\left(z_{j}\right))+\;{\alpha_{3}D}_{KL}\sum_{j}((q_{\phi }\left(z_{j}\right)∥p\left(z_{j}\right)),$$

wheren is a uniform random variable on {1, 2, …, N} with which we relate to data points, the *α*_1_, *α*_2_, and *α*_3_ are hyperparameters. ${ℒ}_{\mathrm{Reconstruction}}$ is the same as the ${ℒ}_{\mathrm{Reconstruction}}$ term in Eq. (4). $p\left(z\right)$ is a prior distribution (Standard normal distribution). $q_{\phi }\left(z\right)$ was calculated by Minibatch stratified sampling.^[20]^ And j indicates a dimensional index of the latent vector z . The large scale of ***α*_2_** makes VAE latent variables independent. We empirically selected ***α***_***1***_=0.75 an *α_3_*=0.75 as the best parameters at the presented loss function. Additionally, we decrease the scale of *α*_2_ from 1.25 to 0.75 by 0.1 per an epoch. *β* at Eq. (4) indicates *α_1_*, *α*_2_, and *α*_3_, and they were determined using the presented formula. Although *β*‐TCVAE increases the independence between latent variables, it may linearly embed the property that is used for metric learning into one latent variable with high variance and scale at the early steps of learning. Therefore, we set γ in Eq. (4) to the following values as a penalty coefficient.

$$\gamma =0.1(1.0-\left(\max_{i}|Pc(z_{i};y\right)|),$$

where *z_i_* are latent vectors that are calculated by the VAE encoder $q_{\phi }\left(z|x\right)$ with a minibatch sample, and i indicates a dimensional index of the latent vector. *y* is a physical property value that is used for metric learning. *P_c_*($z_{i};y)$ represents the Pearson correlation coefficient between the two variables. This penalty term prevents the presented problem. The other VAE parameters are set to the same values as Kajino's MHG‐VAE. As a Joint VAE model f : ${\mathbb{R}}_{50}\to \mathbb{R}$ , we employ a 2‐layer linear regression. The detail list of the setup and benchmark times are listed in the section S3 of the Supplementary Information. The Kajino's MHG‐VAE model can be downloaded from the GitHub page at https://github.com/ibm‐research‐tokyo/graph_grammar. Our model is also available at https://github.com/daiki‐ko/Metric_MHG‐VAE .

## Conflict of Interest

None declared.

## Supporting information

As a service to our authors and readers, this journal provides supporting information supplied by the authors. Such materials are peer reviewed and may be re‐organized for online delivery, but are not copy‐edited or typeset. Technical support issues arising from supporting information (other than missing files) should be addressed to the authors.

Supplementary
