# Supplementary material for: Embedding of Molecular Structure Using Molecular Hypergraph Variational Autoencoder with Metric Learning
Source: Mol Inform. 2020 Nov 23;40(2):2000203. doi: 10.1002/minf.202000203 (PMC7900996; doi:10.1002/minf.202000203)
Supplement: Supplementary file 1 — Supplementary [file MINF-40-2000203-s001.pdf]

molecular informatics

Supporting Information

Embedding of Molecular Structure Using Molecular Hypergraph Variational Autoencoder with Metric Learning

Daiki Koge, Naoaki Ono,* Ming Huang, Md. Altaf-Ul-Amin, and Shigehiko Kanaya©2020 The Authors. Published by Wiley-VCH GmbH. This is an open access article under the terms of the Creative Commons Attribution License, which permits use, distribution and reproduction in any medium, provided the original work is properly cited.

Supporting information for

Embedding of Molecular Structure Using Molecular Hypergraph Variational Autoencoder with Metric Learning

Daiki Koge, Naoaki Ono* , Ming Huang, Md. Altaf-Ul-Amin and Shigehiko Kanaya

Corresponding author e-mail : nono@is.naist.jp

■ Section 1. All 83 properties of RDKit descriptors.

MaxEStateIndex

MinEStateIndex

MaxAbsEStateIndex

MinAbsEStateIndex

qed

MolWt

HeavyAtomMolWt

ExactMolWt

MaxPartialCharge

MinPartialCharge

MaxAbsPartialCharge

MinAbsPartialCharge

FpDensityMorgan1

FpDensityMorgan2

FpDensityMorgan3

BalabanJ

BertzCT

Chi0

Chi0n

Chi0v

Chi1

Chi1n

Chi1v

Chi2n

Chi2v

Chi3n

Chi3v
Chi4n
Chi4v
HallKierAlpha
Ipc
Kappa1
Kappa2
Kappa3
LabuteASA
PEOE_VSA1
PEOE_VSA10
PEOE_VSA11
PEOE_VSA12
PEOE_VSA13
PEOE_VSA14
PEOE_VSA2
PEOE_VSA3
PEOE_VSA4
PEOE_VSA5
PEOE_VSA6
PEOE_VSA7
PEOE_VSA8
PEOE_VSA9
SMR_VSA1
SMR_VSA10
SMR_VSA2
SMR_VSA3
SMR_VSA4
SMR_VSA5
SMR_VSA6
SMR_VSA7
SMR_VSA9
SlogP_VSA1
SlogP_VSA10
SlogP_VSA11
SlogP_VSA2

SlogP_VSA3

SlogP_VSA4

SlogP_VSA5

SlogP_VSA6

SlogP_VSA8

TPSA

EState_VSA1

EState_VSA10

EState_VSA2

EState_VSA3

EState_VSA4

EState_VSA5

EState_VSA6

EState_VSA7

EState_VSA8

EState_VSA9

VSA_EState8

VSA_EState9

FractionCSP3

MolLogP

MolMR

■ Section 2. RMSD, MAE or R2 prediction powers for each of the calculated properties.

For QM9 properties.

Table S1. Comparison of MSE with the models for QM9 data sets. The numbers highlighted in bold show that the model is better. The label indicates the physical property used for embedding learning. Joint and Metric represent the existing and proposed methods, respectively

Label	Model	μ	α	HOMO	LUMO	$\Delta\mathcal{E}$	ZPVE	R^2	U_0	C_v
U_0	Joint	$\mathbf{1.679\times10^{0}}$	1.611×10^{1}	2.477×10^{-4}	$\mathbf{4.159\times10^{-4}}$	$\mathbf{6.713\times10^{-4}}$	1.067×10^{-4}	9.564×10^{3}	1.534×10^{2}	2.769×10^{0}
	Metric	1.851×10^{0}	$\mathbf{7.661\times10^{0}}$	$\mathbf{2.005\times10^{-4}}$	4.822×10^{-4}	7.293×10^{-4}	$\mathbf{9.334\times10^{-5}}$	$\mathbf{8.328\times10^{3}}$	$\mathbf{6.581\times10^{1}}$	$\mathbf{1.747\times10^{0}}$
C_v	Joint	$\mathbf{1.838\times10^{0}}$	9.846×10^{0}	$\mathbf{2.023\times10^{-4}}$	3.461×10^{-4}	5.455×10^{-4}	9.458×10^{-5}	$\mathbf{7.096\times10^{3}}$	1.934×10^{2}	$\mathbf{1.255\times10^{0}}$
	Metric	1.850×10^{0}	$\mathbf{8.495\times10^{0}}$	2.263×10^{-4}	$\mathbf{2.708\times10^{-4}}$	$\mathbf{4.638\times10^{-4}}$	$\mathbf{6.743\times10^{-5}}$	8.446×10^{3}	$\mathbf{1.815\times10^{2}}$	1.340×10^{0}
HOMO	Joint	1.939×10^{0}	1.086×10^{1}	1.746×10^{-4}	3.523×10^{-4}	4.266×10^{-4}	9.416×10^{-5}	$\mathbf{7.022\times10^{3}}$	2.242×10^{2}	1.780×10^{0}
	Metric	$\mathbf{1.277\times10^{0}}$	$\mathbf{1.034\times10^{1}}$	$\mathbf{1.131\times10^{-4}}$	$\mathbf{2.563\times10^{-4}}$	$\mathbf{3.620\times10^{-4}}$	$\mathbf{9.273\times10^{-5}}$	8.216×10^{3}	$\mathbf{1.633\times10^{2}}$	$\mathbf{1.274\times10^{0}}$

Table S2. Comparison of MSE with the models for QM9 data sets.

Label	Model	μ	α	HOMO	LUMO	$\Delta\mathcal{E}$	ZPVE	R^2	U_0	C_v
U_0	Joint	**1.296×10^{0}**	4.014×10^{0}	1.574×10^{-2}	**2.039×10^{-2}**	**2.591×10^{-2}**	1.033×10^{-2}	9.780×10^{1}	1.238×10^{1}	1.664×10^{0}
	Metric	1.361×10^{0}	**2.768×10^{0}**	**1.416×10^{-2}**	2.196×10^{-2}	2.701×10^{-2}	**9.661×10^{-3}**	**9.126×10^{1}**	**8.113×10^{0}**	**1.322×10^{0}**
C_v	Joint	**1.356×10^{0}**	3.138×10^{0}	**1.422×10^{-2}**	1.860×10^{-2}	2.336×10^{-2}	9.725×10^{-3}	**8.424×10^{1}**	1.391×10^{1}	**1.120×10^{0}**
	Metric	1.360×10^{0}	**2.915×10^{0}**	1.504×10^{-2}	**1.645×10^{-2}**	**2.154×10^{-2}**	**8.211×10^{-3}**	9.190×10^{1}	**1.347×10^{1}**	1.158×10^{0}
HOMO	Joint	1.393×10^{0}	3.296×10^{0}	1.321×10^{-2}	1.877×10^{-2}	2.065×10^{-2}	9.704×10^{-3}	**8.380×10^{1}**	1.497×10^{1}	1.334×10^{0}
	Metric	**1.130×10^{0}**	**3.216×10^{0}**	**1.064×10^{-2}**	**1.601×10^{-2}**	**1.903×10^{-2}**	**9.630×10^{-3}**	9.064×10^{1}	**1.278×10^{1}**	**1.129×10^{0}**

Table S3. Comparison of R2 with the models for QM9 data sets.

Label	Model	μ	α	HOMO	LUMO	$\Delta\mathcal{E}$	ZPVE	R^2	U_0	C_v
U_0	Joint	**6.173×10^{-2}**	7.303×10^{-1}	5.000×10^{-1}	**7.675×10^{-1}**	**6.432×10^{-1}**	8.816×10^{-1}	8.739×10^{-1}	8.991×10^{-1}	8.140×10^{-1}
	Metric	1.932×10^{-2}	**8.647×10^{-1}**	**5.182×10^{-1}**	7.346×10^{-1}	6.245×10^{-1}	**8.893×10^{-1}**	**8.784×10^{-1}**	**9.555×10^{-1}**	**8.661×10^{-1}**
C_v	Joint	**1.241×10^{-1}**	8.076×10^{-1}	**5.453×10^{-1}**	8.085×10^{-1}	7.335×10^{-1}	8.875×10^{-1}	**8.875×10^{-1}**	8.487×10^{-1}	9.083×10^{-1}
	Metric	6.907×10^{-2}	**8.466×10^{-1}**	5.384×10^{-1}	**8.496×10^{-1}**	**7.613×10^{-1}**	**9.265×10^{-1}**	8.857×10^{-1}	**8.615×10^{-1}**	**9.098×10^{-1}**
HOMO	Joint	4.928×10^{-3}	7.875×10^{-1}	5.764×10^{-1}	8.079×10^{-1}	7.737×10^{-1}	**8.899×10^{-1}**	**9.005×10^{-1}**	8.252×10^{-1}	8.695×10^{-1}
	Metric	**2.674×10^{-1}**	**8.037×10^{-1}**	**7.425×10^{-1}**	**8.580×10^{-1}**	**8.031×10^{-1}**	8.895×10^{-1}	8.804×10^{-1}	**8.681×10^{-1}**	**9.010×10^{-1}**

For RDKit Descriptors.

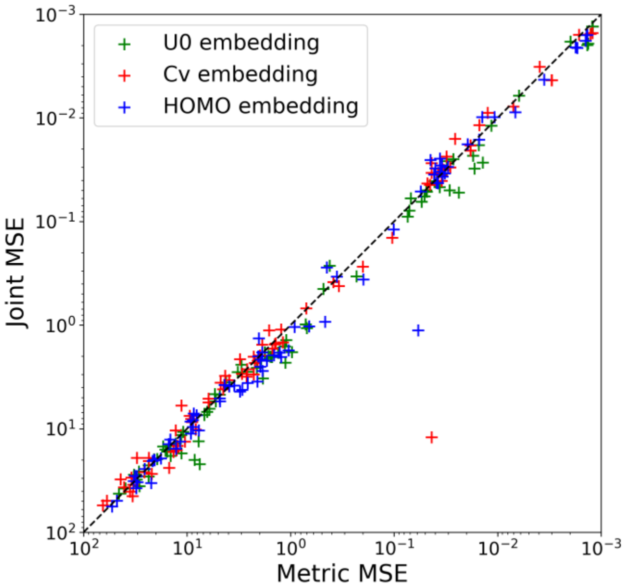

Figure S1. MSE score comparison of Joint and Metric VAEs (our proposed model) broken down by RDKit descriptors and embedding labels. Each dot represents RDKit descriptors, while each marker color represents a physical property value used in the embedding learning model.

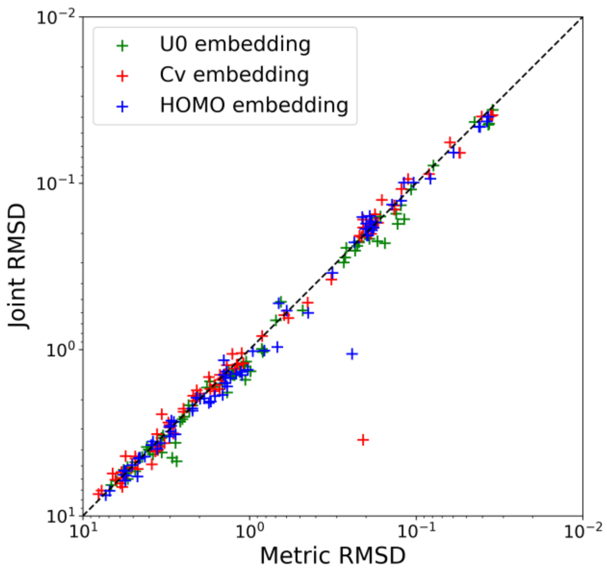

Figure S2. RMSD score comparison of Joint and Metric VAEs (our proposed model) broken down by RDKit descriptors and embedding labels. Each dot represents RDKit descriptors, while each marker color represents a physical property value used in the embedding learning model.

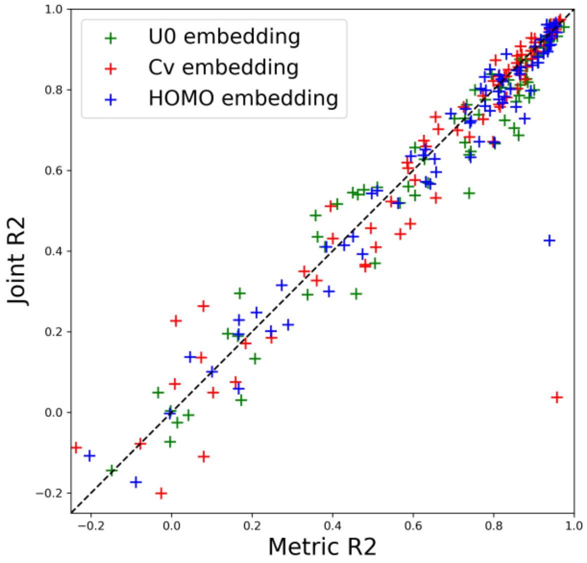

Figure S3. R2 score comparison of Joint and Metric VAEs (our proposed model) broken down by RDKit descriptors and embedding labels. Each dot represents RDKit descriptors, while each marker color represents a physical property value used in the embedding learning model.

Section 3. Details on computer setup and CPU and GPU specifications and computational times.

- OS

Ubuntu 16.04.6 LTS

- CPU

product: Intel(R) Xeon(R) CPU E5-1650 v4 @ 3.60GHz

vendor: Intel Corp.

size: 3599MHz

capacity: 4GHz

width: 64 bits

- GPU

product : GP104 [GeForce GTX 1080]

vendor : NVIDIA Corporation

This code for this study was tested in Python 3.6.6 with Pytorch 0.4.1.

- Computational time with GPU

Mode	Sample size	Batch size	Epoch time
Train	99968	64	3002.102 (sec)
Validation (inference)	28928	64	578.184 (sec)
